# Interleukin 13 and Serotonin: Linking the Immune and Endocrine Systems in Murine Models of Intestinal Inflammation

**DOI:** 10.1371/journal.pone.0072774

**Published:** 2013-08-28

**Authors:** Md. Sharif Shajib, Huaqing Wang, Janice J. Kim, Ivana Sunjic, Jean-Eric Ghia, Emmanuel Denou, Matthew Collins, Judah A. Denburg, Waliul I. Khan

**Affiliations:** 1 Farncombe Family Digestive Health Research Institute, Hamilton, Ontario, Canada; 2 Department of Pathology & Molecular Medicine, McMaster University, Hamilton, Ontario, Canada; 3 Department of Immunology, University of Manitoba, Winnipeg, Canada; 4 Department of Medicine, McMaster University, Hamilton, Ontario, Canada; 5 Hamilton Regional Laboratory Medicine Program, Hamilton Health Sciences, Hamilton, Ontario, Canada; University Heart Center Freiburg, Germany

## Abstract

**Objective:**

Infiltration of activated immune cells and increased cytokine production define the immunophenotype of gastrointestinal (GI) inflammation. In addition, intestinal inflammation is accompanied by alteration in the numbers of serotonin (5-hydroxytryptamine; 5-HT) synthesizing enterochromaffin (EC) cells and in 5-HT amount. It has been established that EC cells express interleukin (IL)-13 receptor, additionally IL-13 has been implicated in the pathogenesis of ulcerative colitis. In this study, we investigated the role of IL-13 mediated 5-HT signaling in pathogenesis of colitis.

**Methodology:**

Colitis was induced in IL-13 deficient (IL-13−/−) and wild-type (WT) mice with dextran sulfate sodium (DSS) and dinitrobenzene sulfonic acid (DNBS), as well as in IL-13−/− mice given recombinant mouse IL-13 (rmIL-13) and 5-hydroxytryptamine (5-HTP), the direct precursor of 5-HT.

**Principal Findings and Conclusion:**

Elevated colonic IL-13 levels were observed in WT mice receiving DSS in comparison to control. IL-13−/− mice administered DSS exhibited significantly reduced severity of colitis compared to WT mice as reflected by macroscopic and histological damage assessments. Following DSS administration, significantly lower pro-inflammatory cytokine production and fewer infiltrating macrophages were observed in IL-13−/− mice compared to WT. The reduced severity of colitis observed in IL-13−/− mice was also accompanied by down-regulation of EC cell numbers and colonic 5-HT content. In addition, increasing colonic 5-HT content by administration of rmIL-13 or 5-HTP exacerbated severity of DSS colitis in IL-13−/− mice. IL-13−/− mice also exhibited reduced severity of DNBS-induced colitis. These results demonstrate that IL-13 plays a critical role in the pathogenesis of experimental colitis and 5-HT is an important mediator of IL-13 driven intestinal inflammation. This study revealed important information on immune-endocrine axis in gut in relation to inflammation which may ultimately lead to better strategy in managing various intestinal inflammatory conditions including inflammatory bowel disease.

## Introduction

Intestinal inflammation is characterized by mucosal recruitment of activated cells from both the innate and adaptive arms of the immune system; [1] this is exemplified in both Crohn’s disease (CD) and ulcerative colitis (UC). CD and UC have distinct immunophenotypes. [2] Whereas CD is due to a T helper (Th)1/Th17 type response, UC is considered to be an atypical Th2 disease. [2], [3] Increased levels of interleukin (IL)-13 and IL-5, but not IL-4, have been observed in association with UC and recently, it was identified that a non-invariant subset of natural killer T (NKT) cells are the source of this increased IL-13 production in these patients. [2], [4], [5] Moreover, targeted inhibition of IL-13 by interferon (IFN)-β-1a yielded promising results in UC patients responsive to the treatment, further implicating IL-13 in the pathogenesis of UC. [6]. This notion is supported by findings in animal model of UC, namely the oxazolone model. [7], [8] These studies reflect the great strides made in mucosal immunology regarding the immunopathogenesis of CD and UC. However, the effects of the distinct immunophenotypes of these inflammatory conditions on the coexisting entero-endocrine system within the gastrointestinal (GI) tract are yet to be fully understood.

The entero-endocrine system is a collection of specialized epithelial cells that establishes the GI tract as the largest endocrine system in the human body. [9] Enterochromaffin (EC) cells are the best characterized enteric endocrine cells and are the main source of serotonin (5-hydroxytryptaime; 5-HT) in the GI tract. 5-HT is considered to be an important enteric mucosal signalling molecule with vital roles in the physiology and inflammatory conditions of the gut. [10] Changes in EC cell numbers and 5-HT content have been associated with various GI disorders, including CD and UC. [11] Similarly, alterations in normal 5-HT signalling has been reported in various animal models of intestinal inflammation, [1], [12], [13] and given the strategic location of EC cells in the gut mucosa, [14] it is likely that they are mediators of the cross-talk between the immune and the entero-endocrine systems. Providing evidence for immune regulation of EC cell biology, we have shown that 5-HT production in the gut can be regulated by CD4+ T cells, and EC cell numbers and colonic 5-HT content differ in different cytokine milieu. [15], [16] EC cells express IL-13 receptor alpha1-chain (IL-13Rα1); in addition, IL-13 has been shown to up-regulate tryptophan hydroxylase (TPH)-1 protein expression and 5-HT production, both in an *in vivo* model of enteric infection and *in vitro* using a model for human EC cells [16], [17].

In view of the critical role of 5-HT signalling in the generation of intestinal inflammation, [10], [18] and the connection of IL-13 in pathogenesis of IBD, specifically of UC, in the present study we investigated the role of IL-13 in the pathogenesis of colitis in the context of gut 5-HT production in two different models of experimental colitis [dextran sodium sulfate (DSS) and dinitrobenzenesulfonic acid (DNBS)]. We hypothesized that the IL-13-EC cell/5-HT axis is important in the pathogenesis of colitis whereby manipulation of the immune system to alter IL-13 production will modulate 5-HT production as well as the severity of colitis. Our study revealed an important role of IL-13 in the generation of intestinal inflammation in relation to 5-HT production and demonstrated that IL-13 gene deficient mice, which have less 5-HT, are better protected against these models of inflammation, and *in vivo* replenishment of 5-HT abrogates these benefits. These results identified 5-HT as an important mediator of IL-13 driven intestinal inflammation.

## Materials and Methods

### Ethics Statement

Animal protocols used for the study were in accordance with McMaster University Animal Care Committee and guidelines set by Canadian Council of the Use of Laboratory Animals. The protocols were approved by Animal Research Ethics Board (AREB)-McMaster University (AUP #11-04-16).

### Animals

Male IL-13 deficient mice on BALB/c background (IL-13−/−) [19] (gift from Dr. Manel Jordan, McMaster University, Hamilton, ON) and age matched wild-type (WT) counterparts were used for the purposes of these experiments. All mice were kept in sterilized, filter-topped cages, fed autoclaved food at a temperature of 21–22°C and with 12 h light and 12 h dark cycle in the McMaster University central animal facility. The animals were allowed to acclimatize for 1 week prior to the start of any experiments.

### Induction of DSS and DNBS Colitis

DSS (mol wt. 36-54 kilodaltons: ICN Biomedicals Inc, Soho, OH) was added to drinking water at 5% weight/volume for five days. The average DSS consumption per cage was recorded every day for the duration of the experiment. For induction of DNBS colitis, mice were anesthetized with isoflurane (Abbott, Toronto, Canada) prior to intrarectal (IR) instillation of DNBS (3 mg in 100 µL of 50% ethanol; ICN Biomedicals Inc.), or 50% ethanol as vehicle, via a 10-cm-long PE-90 tubing (ClayAdam, Parsippany, NJ), attached to a tuberculin syringe, inserted 3.5 cm into the colon. All groups were supplied with 6% sucrose in drinking water to prevent dehydration.

### Experimental Design

DSS colitis was induced by orally administering 5% DSS in drinking water for 5 days. In a separate experiment, IL-13−/− mice were injected subcutaneously with 100 mg/kg of 5-hydroxytryptophan (5-HTP) (Sigma–Aldrich, Mississauga, Canada) twice daily for 8 days beginning 3 days prior to induction of DSS colitis; whereas, the control IL-13−/− mice received saline as vehicle. In another experiment, IL-13−/− mice received recombinant mouse (rm)IL-13 (R&D Systems, Minneapolis, MN) at a dose of 2 µg/day, starting 3 days before a 5 day period of DSS administration. In DNBS colitis, mice were euthanized 3 days post-DNBS administration. For all experiments, animals were anaesthetized prior to euthanization via cervical dislocation at the conclusion of each experiment or if they reached a predetermined end point (ie, loss of ≥20% body weight and/or significant deterioration of body condition).

### Evaluation of Inflammation

For the duration of all experiments, the weights of the mice were recorded daily, and were expressed as a percentage of body weight prior to induction of colitis. Percentage of body weight lost in combination with stool consistency and feces bleeding comprised the disease activity index (DAI). [20] Macroscopic scoring was performed immediately after the mice were sacrificed using previously established scoring system for DSS and DNBS. [20], [21] Categories evaluated for DSS macroscopic scores included, rectal bleeding, rectal prolapse, diarrhea and colonic bleeding, whereas the DNBS macroscopic scores reflect numerical values assigned to the categories, adhesion, hyperaemia, thickness, fecal consistency, number of ulcers and sizes. For the purposes of histological scoring, colonic segments collected during sacrifice were fixed in 10% phosphate buffered formalin and stained with H&E. Colonic damage was assessed based on a published scoring system that considered loss of architectural, degree of inflammatory cell infiltrate, goblet cell depletion, muscle thickening and crypt abscess. [20], [21] Myeloperoxidase (MPO) activity was determined using a previously published method and is expressed as unit per mg of tissue [22].

### Colonic Immunohistochemistry and Peritoneal Macrophage Culture

Immunohistochemical studies of Ki-67 positive cells, 5-HT expressing EC cells and F4/80 positive macrophages were performed on paraffin-wax-embedded colonic sections, as previously described. [1], [10], [15], [23] Primary antibody used for Ki-67 immunohistochemisrty was a rabbit anti-Ki67 antibody (Abcam, Cambridge, UK), for 5-HT was a rabbit anti-5-HT antibody (Immunostar, Hudson, WI) and for F4/80 was a rat anti-mouse F4/80 antibody (AbD Serotec, Kidlington, UK). Immunostained sections were observed at 200× magnification and the number of Ki-67 positive cells, 5-HT positive EC cells were expressed as number of positive cells per 1 and 10 gland, respectively, and F4/80 positive macrophage areas were quantified using ImageJ (National Institutes of Health, Bethesda, MD) and expressed as a percent of total area.

For macrophage culture, peritoneal cavity cells were collected from WT and IL-13−/− mice with or without DSS treatment and cultured as described previously. [10] Cells were plated at a concentration of 3.0× 10^6^ cells per millilitre, and treated with either lipopolysaccharides (LPS); (100 ng/mL; Sigma-Aldrich) or 5-HT (10^−10^ mol/L; Sigma-Aldrich) or both for 24 hours. The culture supernatant were collected and stored in -80°C until determination of cytokine levels using Bioplex protein array system (Bio-Rad Inc, Hercules, CA).

### Serum IL-13 and Tissue Cytokine Levels

Blood was collected via intra-cardiac puncture from anesthetized (isoflurane) mice following five and eight days of DSS and rmIL-13 treatment, respectively. Colonic samples were homogenized in 1 ml of Tris HCl buffer containing protease inhibitor cocktail (Sigma-Aldrich). Samples were centrifuged and the supernatant was collected and frozen at –80°C until assay. Cytokine levels in tissue and serum were determined using commercial available enzyme-linked immunosorbent assay (ELISA) kit (R&D Systems).

### Determination of Colonic 5-HT Levels

Colonic 5-HT levels were determined for all *in vivo* experiments as previously described. [15] Briefly, colonic tissues were weighed and were homogenized in 0.2 N perchloric acid. Following centrifugation at 10,000 g for 5 minutes, the supernatant was collected and the pH was neutralized using 1 M borate buffer. The supernatant was stored in -80°C until analysis of 5-HT levels using commercially available ELISA kits (Beckman Coulter, Fullerton, CA). 5-HT content was expressed as a function of wet weight (ng/mg).

### Statistical Analysis

All statistical analysis was performed using GraphPad Prism 5 (GraphPad Software, San Diego, CA) and results are represented as mean or percentage of control. Student’s t-test and one way ANOVA followed by the Newman-Keuls multiple comparison test were used where appropriate and a p-value of <0.05 was considered statistically significant.

## Results

### IL-13 Deficiency is Associated with Decreased 5-HT Production and Severity of DSS Colitis

**Figure 1:**
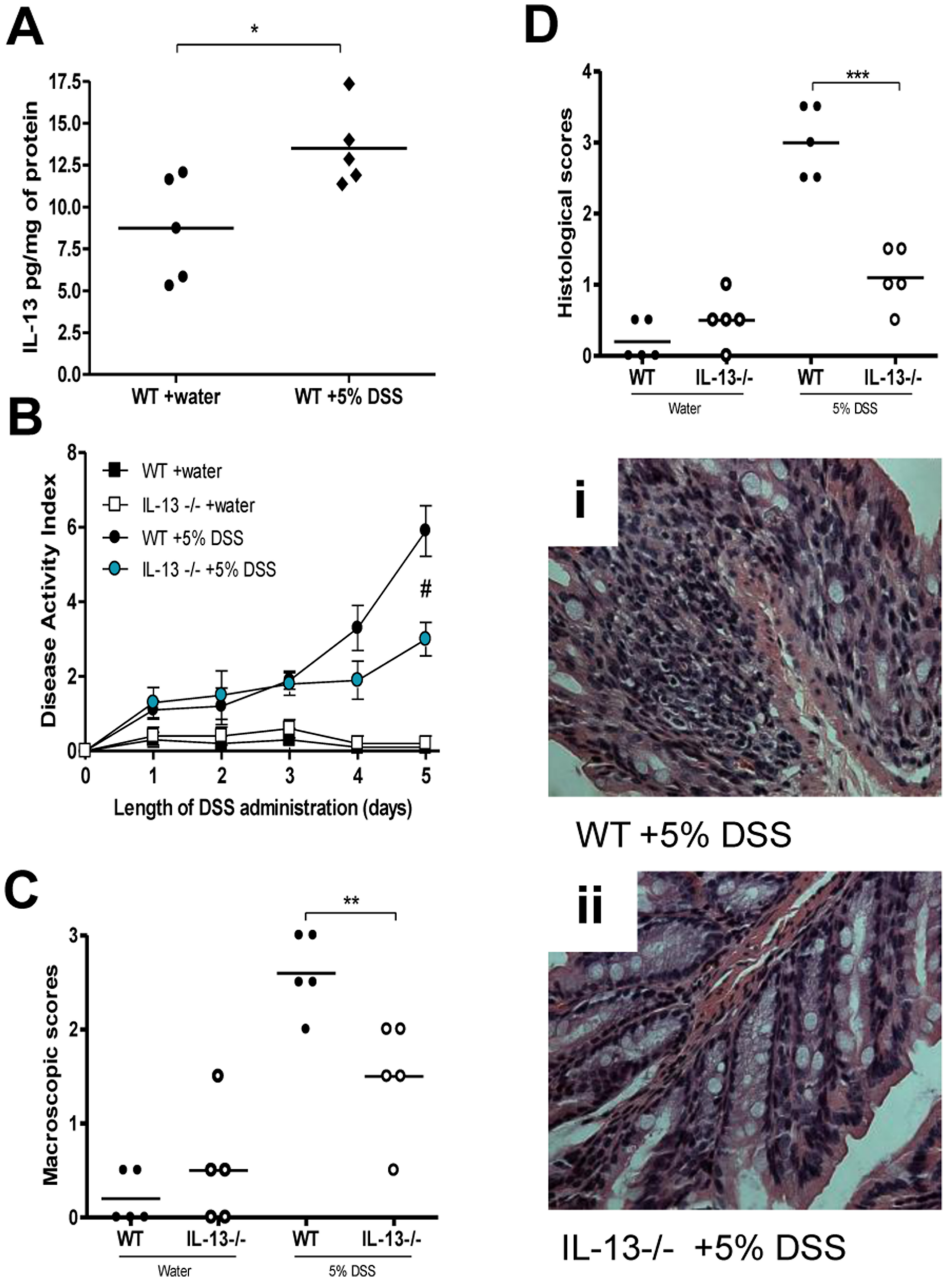
Effects of IL-13 deficiency in DSS-induced colitis. WT and IL-13−/− mice were administered 5% DSS in drinking water to induce colitis. Control mice received water without DSS. (A) Colonic IL-13 levels in WT mice with or without DSS. (B) Disease activity index (DAI). (C) Macroscopic damage score in DSS-induced colitis on day 5 after DSS induced colitis and in mice without colitis. (D) Histological damage assessment on day 5 post-DSS administration. (i) And (ii) Light micrograph of H&E-stained colonic section. DAI data represented as mean ± SEM from 5 mice per group; *represents statistical significance where p<0.05; ^#^ significantly lower disease activity in IL-13−/− mice receiving DSS compared to WT mice receiving DSS.

**Figure 2:**
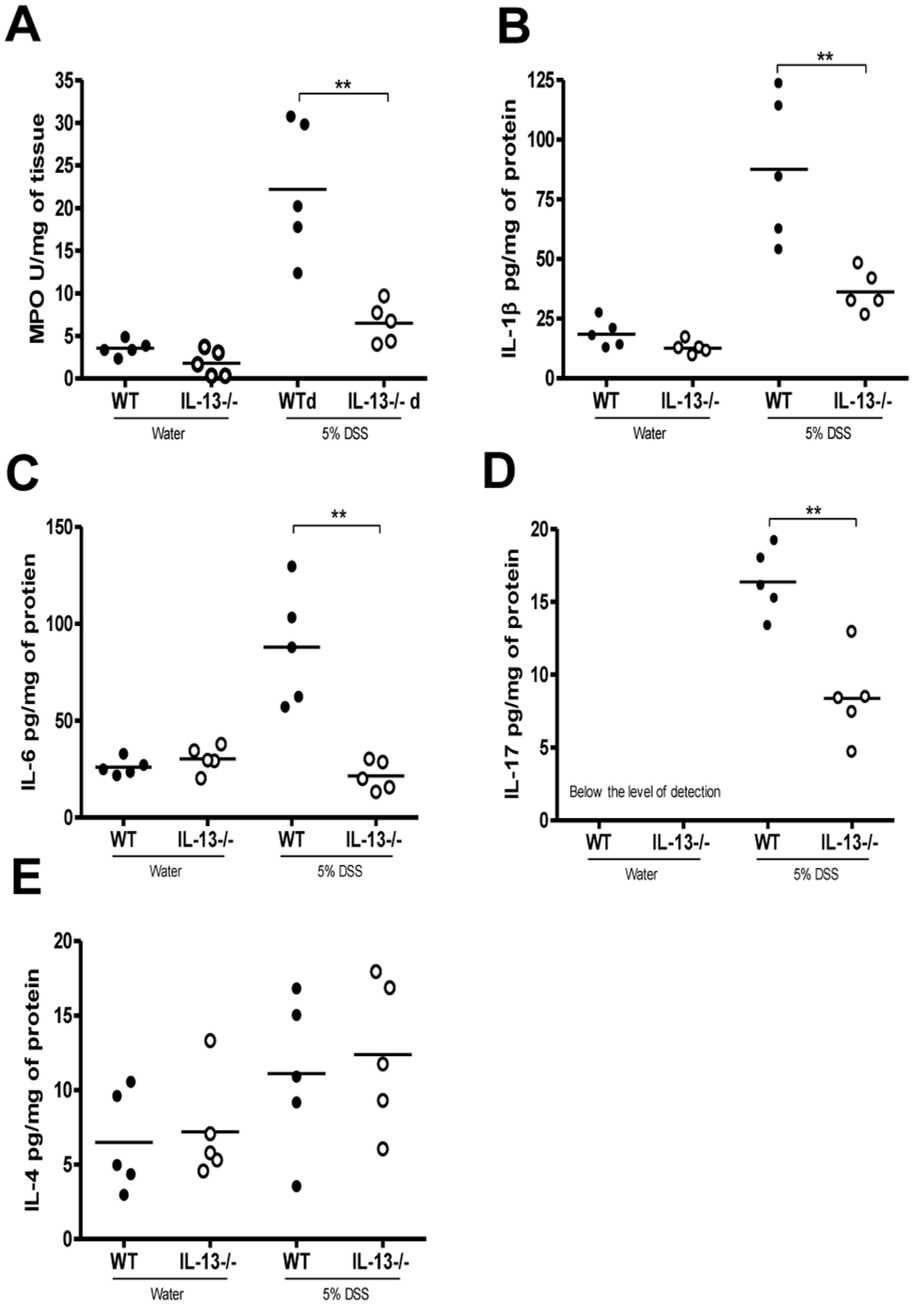
Effects of IL-13 deficiency on MPO activity and colonic cytokine levels in DSS-induced colitis. WT and IL-13−/− mice were given 5% DSS in drinking water to induce colitis and were sacrificed on day 5 post-DSS administration. (A) MPO activity, (B) IL-1β (C) IL-6 (D) IL-17 and (E) IL-4 levels in colonic tissues. *Represents statistical significance where p<0.05.

Colonic IL-13 levels are significantly increased in WT mice on day 5 post-DSS as compared to controls without DSS (Figure 1A). IL-13−/− mice exhibited significantly reduced disease activity compared to WT mice post-DSS administration (Figure 1B). Reduced disease activity was consistently observed in IL-13−/− mice, compared to WT mice, regardless of litters, cages and time of the experiment (Figure S1). IL-13−/− mice also had marked reductions in macroscopic and histological damage scores (Figure 1C and D). Reduction in the severity of DSS colitis was associated with significantly lower MPO activity, as well as reduced IL-1β, IL-6 and IL-17 production in IL-13−/− mice (Figure 2A–D). However, IL-4 levels observed were not significantly different amongst any of the groups (Figure 2E).

**Figure 3:**
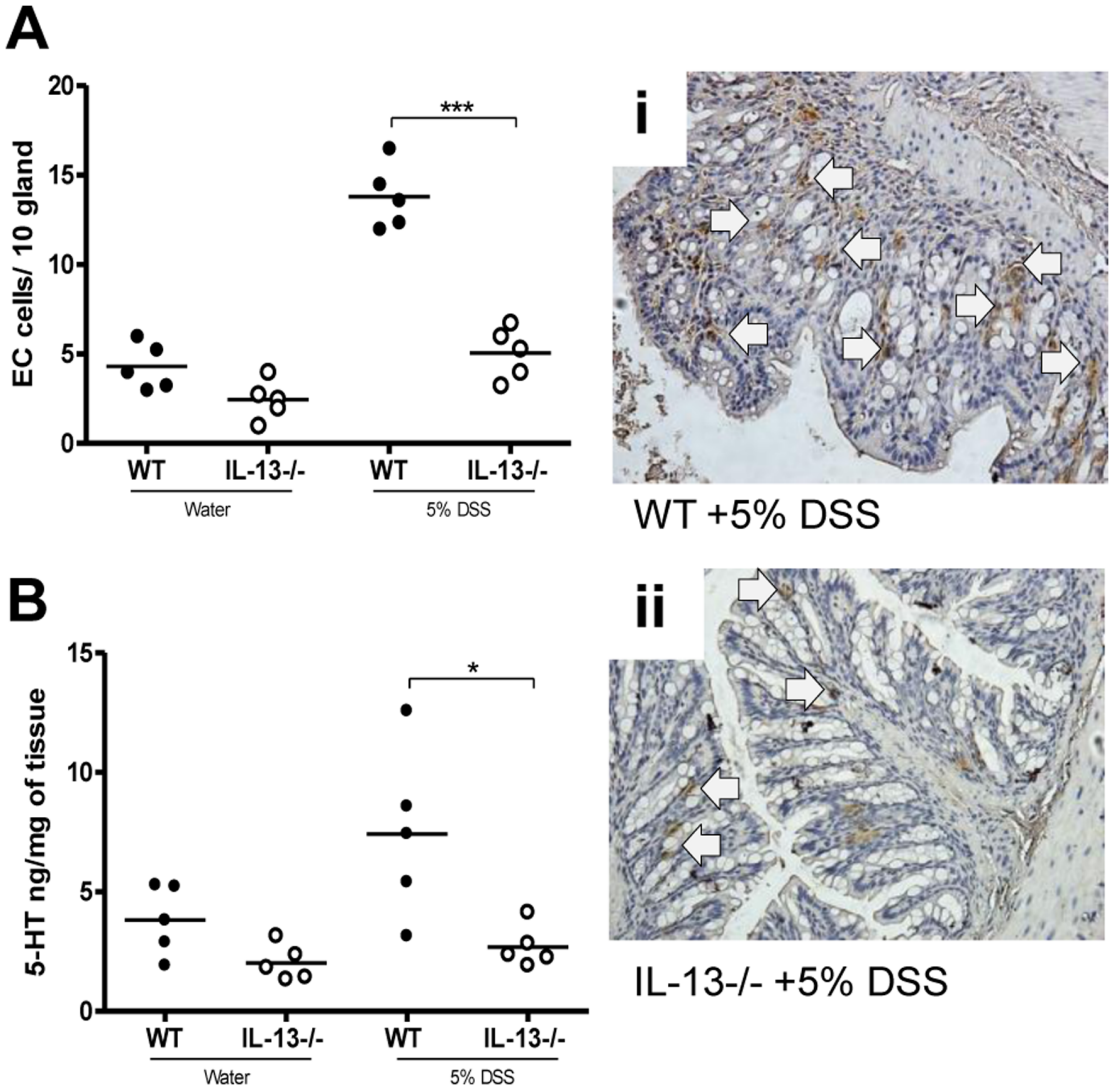
Effects of IL-13 deficiency in DSS-induced colitis. WT and IL-13−/− mice were given 5% DSS in drinking water to induce colitis. (A) Number of 5-HT expressing EC cells per 10 glands and colonic sections immunostained for 5-HT expressing EC cells after 5 days of DSS administration in WT and IL-13−/− mice. (B) Colonic 5-HT amount. (i) and (ii) Representative micrograph and arrows indicate 5-HT expressing EC cells. *Represents statistical significance where p<0.05.

In this study, significantly reduced 5-HT expressing EC cell numbers were observed in IL-13−/− mice following induction of DSS colitis compared to WT mice (Figure 3A). There was also reduced colonic 5-HT content in IL-13−/− mice in comparison to WT mice (Figure 3B). Recently we have identified a role of IL-13 in murine EC cell biology and BON cell hyperplasia, BON cells are human carcinoid cells that secrete 5-HT thus used as a model of EC cells. [17] In this study, we observed significantly increased in Ki-67 positive cells in WT mice after induction of DSS colitis in WT mice but this DSS-induced up-regulation of epithelial cell proliferation was not evident in IL-13−/− mice (Figure S2). Taken together, these observations suggest that IL-13 plays an important role in EC cell biology and 5-HT production and in generation of inflammation in DSS colitis.

### Dampened Response in Cytokine Production by Peritoneal Macrophages and Fewer Activated Macrophages in Mice Lacking IL-13 Following DSS Administration

**Figure 4:**
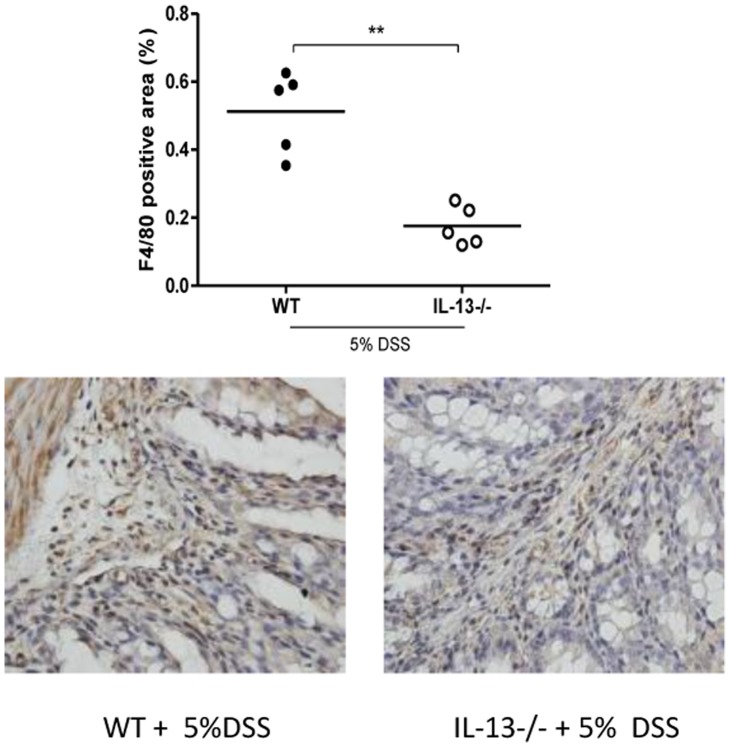
F4/80 positive macrophage infiltration in DSS-induced colitis in WT and IL-13−/− mice. F4/80 positive area, positive stained areas expressed as the percent of the total area. (i) and (ii) Representative micrographs. *Represents statistical significance where p<0.05.

Reduced 5-HT production in the gut has previously been associated with fewer infiltrating F4/80 positive macrophages in experimental colitis. [10] Consequently, the reduced production of 5-HT observed in the IL-13−/− mice was accompanied by fewer infiltrating macrophages in their colonic segments following DSS treatment in comparison with WT mice (Figure 4). Earlier work in our lab established that, 5-HT stimulation increases IL-1β and IL-6 production by peritoneal resident macrophages. [10]. In the current study, it was observed that macrophages isolated from IL-13−/− mice had dampened cytokine production in comparison to WT mice as revealed when normalized to their respective controls (Table 1) and in culture treatment with 5-HT was able to reverse these effects in IL-13−/− macrophages (Table 1).

**Table 1:** Effects of 5-HT on pro-inflammatory cytokine production.

*in vitro* treatment	IL-1β		IL-6	
	WT	IL-13−/−	WT	IL-13−/−
Media	15.53±3.13	12.95±1.59	6.88±0.67	4.67±1.96
5-HT	22.07±1.48	15.26±4.33	12.42±0.93	11.83±1.16

Effects of 5-HT on IL-1β and IL-6 production by peritoneal macrophages isolated from WT and IL-13−/− following DSS administration. Data is expressed as percent increase with respect to their controls; mean ± SEM of 4 to 6 mice in each group done in triplicates.

### Increased Availability of 5-HT Exacerbates Severity of Colitis in IL-13 Deficiency

**Figure 5:**
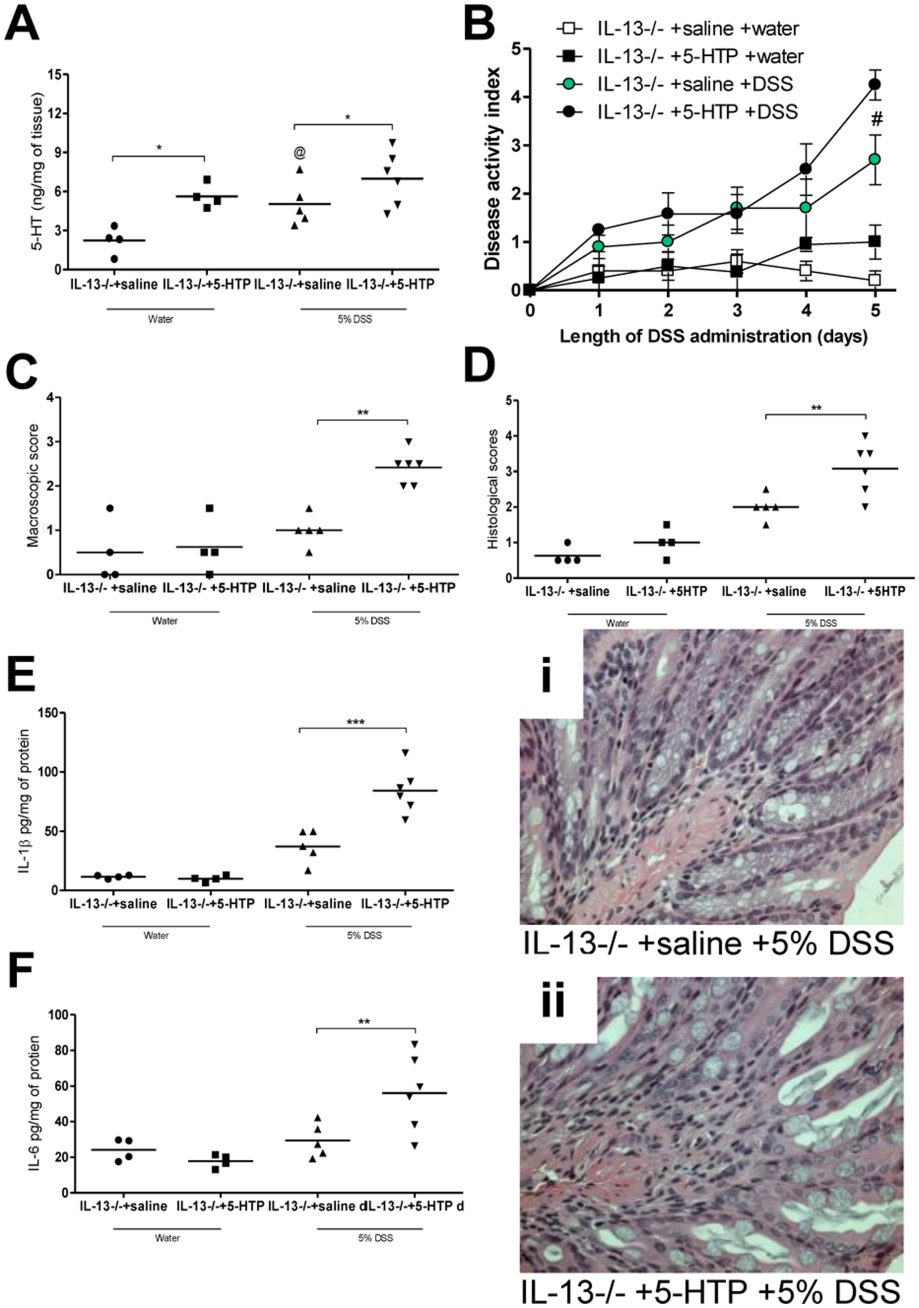
Effects of 5-HTP administration in IL-13−/− mice in DSS-induced colitis. 5-HTP 100 mg/kg treatment started 3 days prior to 5 days of 5% DSS administration, control groups received saline. (A) Colonic 5-HT levels (B) DAI (C) Macroscopic scores (D) Histological scores (i) and (ii) Representative light micrograph of an H&E-stained colonic section. From colonic tissue (E) IL-1β and (F) IL-6 levels. DAI data represented as mean ± SEM from 4 to 6 mice; *represents statistical significance where p<0.05; ^#^significant difference between IL-13−/− +5-HTP and IL-13−/− +saline, both groups receiving DSS; ^@^ indicates no significant difference between IL-13−/− groups receiving saline as vehicle.

To evaluate the role of decreased 5-HT production in IL-13−/− mice and its significance in the reduced severity of DSS-colitis in these animals, we treated IL-13−/− mice with 5-HTP, the direct precursor of 5-HT. A significant increase in colonic 5-HT content was observed in IL-13−/− mice receiving 5-HTP compared to IL-13−/− receiving vehicle following induction of DSS colitis (Figure 5A). IL-13−/− mice treated with 5-HTP had significantly higher disease activity scores compared to the vehicle treated group (Figure 5B). Post-mortem macroscopic assessment and histopathological evaluation also revealed significant enhancement of severity of colitis in IL-13−/− mice that received 5-HTP as compared to the IL-13−/− mice that received vehicle (Figure 5C and D). This increase in severity of colitis observed in 5-HTP treated groups was also resulted in increased levels of colonic IL-1β and IL-6 (Figure 5E and F).

### Replenishment of IL-13 Levels in IL-13−/− Mice Increases Severity of Colitis

**Figure 6:**
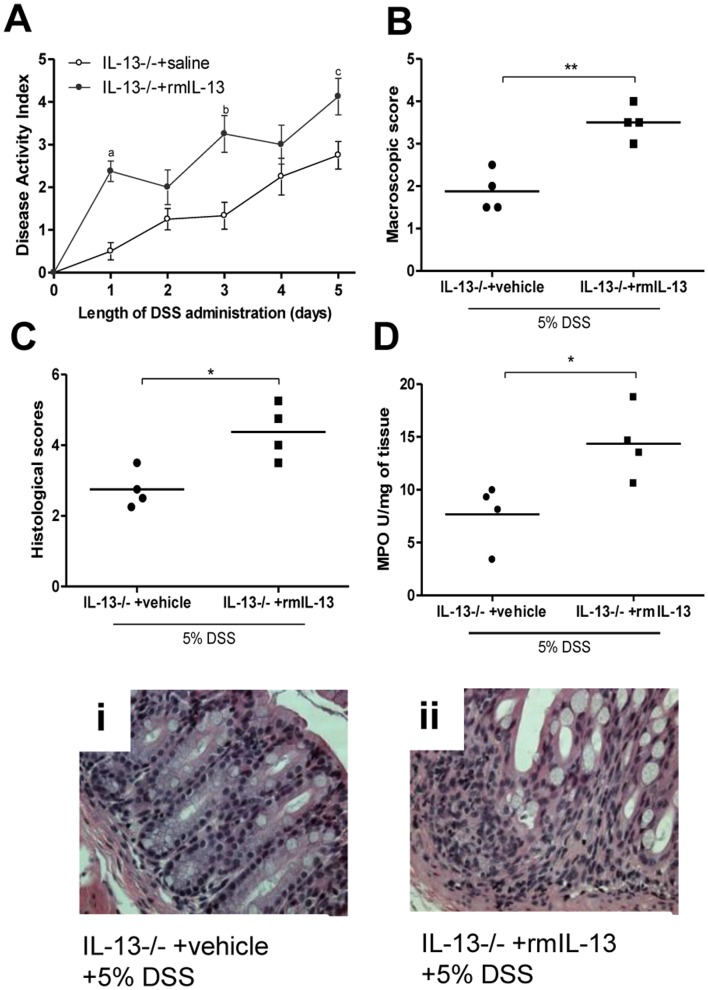
Effects of replenishment of IL-13 in DSS-induced colitis. IL-13−/− mice were treated with rmIL-13 (2 µg/day) injections or vehicle. rmIL-13 treatment started 3 days prior to 5 days of 5% DSS treatment. All groups received 5% DSS in the drinking water to induce colitis. (A) DAI. (B) Macroscopic damage score (C) Histologic scores and (D) MPO activity. (i) and (ii) Representative light micrograph of an H&E-stained colonic section. DAI data represented as mean ± SEM from 4 mice; *represents statistical significance where p<0.05; a, b and c represents statistically significant difference in disease activity scores on days indicated, where p<0.05.

**Figure 7:**
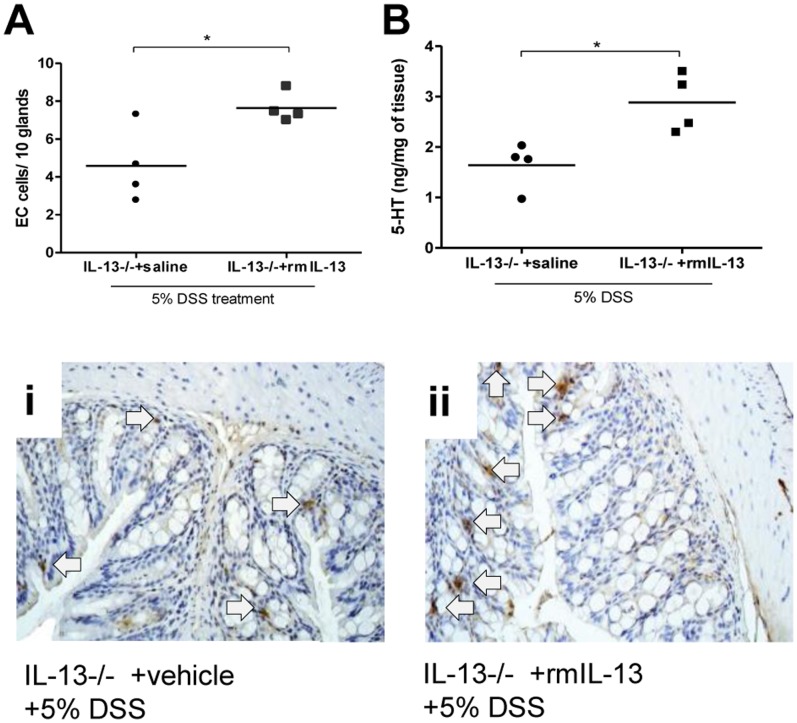
Effects of replenishment of IL-13 in DSS-induced colitis. IL-13−/− mice were treated with rmIL-13 (2 µg/day) injections or vehicle. rmIL-13 treatment started 3 days prior to 5 days of 5% DSS treatment. All groups received 5% DSS in the drinking water to induce colitis. (A) Number of 5-HT expressing EC cells per 10 glands and (B) colonic 5-HT content. (i) and (ii) Representative micrograph and arrows indicate 5-HT expressing EC cells. *Represents statistical significance where p<0.05.

To confirm the effects of IL-13 in 5-HT production and in turn severity of colitis, we reconstituted IL-13−/− mice with rmIL-13. Administration of rmIL-13 significantly increased IL-13 levels in IL-13−/− mice (524.82±120.81 pg/ml) compared to mice receiving vehicle (below the level of detection). Restoration of IL-13 levels in IL-13−/− mice were associated with increased severity of DSS-induced colitis and increased levels of MPO activity (Figure 6A–D). Along with increased severity of colitis, a significant increase in 5-HT expressing EC cell numbers and 5-HT production was observed in the IL-13−/− mice receiving rmIL-13 compared to IL-13−/− mice receiving vehicle (Figure 7 A and B).

### IL-13 Deficiency Associated Down-regulation of 5-HT has Protective Effects in DNBS- Colitis

**Figure 8:**
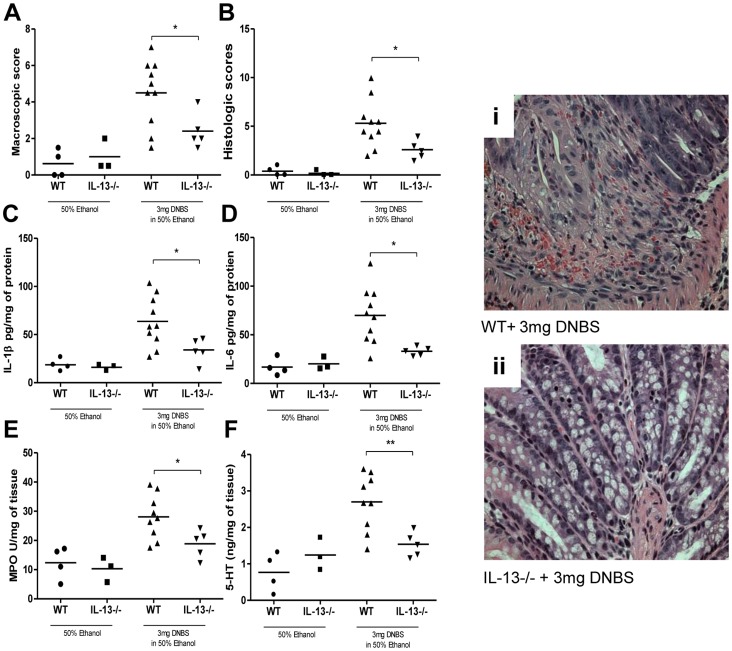
Effects of lack of IL-13 in DNBS-induced colitis. WT and IL-13−/− mice were given DNBS (3 mg/100 µL) in 50% ethanol solution. All controls received 50% ethanol via intrarectal installation. Mice were sacrificed on day 3 following induction of DNBS colitis. (A) Macroscopic scores and (B) Histologic scores. (i) and (ii) Representative micrographs. From colonic tissue (C) IL-1β (D) IL-6 levels (E) MPO activity, and (F) 5-HT levels. *Represents statistical significance where p<0.05.

To investigate whether decreased 5-HT production and reduced severity of colitis observed in IL-13−/− mice was restricted to the DSS model; we induced DNBS colitis in IL-13−/− mice. Post-DNBS administration, we observed significantly reduced macroscopic and histological damage in IL-13−/− mice in comparison to WT mice (Figure 8A and B). There was also significant reduction in colonic IL-1β, IL-6 levels and MPO activity observed in the IL-13−/− mice (Figure 8 C-E). In addition, there was a significant down-regulation of 5-HT production observed in the IL-13−/− mice in comparison to WT mice following DNBS-induced colitis (Figure 8F).

## Discussion

IBD affects approximately 1.4 million people in North America and costs the healthcare system upwards of $2 billion every year. [24] Although the exact etiology of IBD is not known, studies have provided evidence that dysregulated immune response, genetic factors, gut flora, and environmental factors contribute to the pathogenesis of IBD. [25] Alterations in EC cell population, the main source of our body’s 5-HT, have been associated with these disorders. [11] Considering the close proximity between lymphocytes and EC cells in the gut mucosa, it is not farfetched to infer that these cells are the gatekeepers at the critical junction of immuno-endocrine interaction, though the mechanism of how the immune and the endocrine systems influence each other and how their interactions may shape the progression or regression of disease is still not well understood. In the current study, using two different models of experimental colitis, we identified 5-HT as an important arbitrator of IL-13-mediated gut inflammation.

IL-13 is a pleiotropic cytokine that exerts its influence on various cell types, including epithelial cells, macrophages, smooth muscle cells and neurons. [26] A plethora of immune cells produce IL-13, [26] and a subset of NKT cells have been identified as the source of increased IL-13 production in patients with UC. [5] Previously, our lab has shown that, in enteric infection driven intestinal inflammation as well as in human model of EC cells, IL-13 plays a vital role in the up-regulation of the rate-limiting enzyme TPH-1 and in turn 5-HT production. [17] It has also been demonstrated that a Th2 immunophenotype is more apt in the regulation of EC cell biology relative to a Th1 immunophenotype. [15] In this study, for the first time, we report a significant increase in colonic IL-13 levels following DSS administration; this is in line with findings in other models of UC. [8] Using IL-13 deficient mice we observed that these mice suffered from reduced severity of colitis and had significantly fewer 5-HT expressing EC cells and lower 5-HT content in the gut. Traditionally, IL-13 deficiency is not associated with depressed inflammatory cytokines production; [27] we postulated that it is the lower colonic 5-HT content that is responsible for the decreased severity of colitis observed in IL-13−/− mice. This became evident when severity of colitis in IL-13−/− mice increased following enhancement of colonic 5-HT amount via 5-HTP treatment, which was also marked by the increased production of pro-inflammatory cytokines.

5-HT is an important neurotransmitter of the central nervous system and is studied extensively for its role in regulating behavior, appetite and energy expenditure. [28], [29] What is often underappreciated is that the vast majority of 5-HT in the body is found in GI tract not the central nervous system. Recently, our lab has shown that 5-HT plays a key role in generation of inflammation and immune activation. [10], [30] By subjecting TPH-1 deficient (TPH-1−/−) mice, which have significantly reduced colonic 5-HT content, to DSS and DNBS models of colitis, it was demonstrated that the TPH-1−/− group experienced significantly reduced severity colitis and had lower production of pro-inflammatory cytokines. [10] Ghia and colleagues [10] also established the role of 5-HT in recruitment and in the induction of a pro-inflammatory phenotype in macrophages. Given the reduced production of colonic 5-HT observed in the IL-13 deficient group following DSS treatment we anticipated that there would be reduced number of infiltrating macrophages in these animals, and this was confirmed by fewer F4/80 positive macrophages observed in the colonic segments of IL-13−/− mice. In addition, we observed that macrophages isolated from IL-13−/− mice had a dampened response following DSS administration in comparison to WT, and their response was bolstered following in culture 5-HT treatment. Thus, revealing how regulation of 5-HT production by IL-13 may mediate inflammation.

To further explore the role of the IL-13-5-HT axis in severity of DSS-induced colitis, we replenished IL-13 content in IL-13−/− mice and found that this resulted in a significant increase in severity of colitis as reflected by the disease activity index, macroscopic and histological damage assessment. Elevation of IL-13 levels was once again marked by a significant increase in colonic 5-HT content as well as 5-HT expressing EC cell numbers. The increased production of IL-13 and the resulting increase in 5-HT observed in the DSS model of colitis and the role of 5-HT in the other models of colitis previously observed, prompted investigation into the effects of this association in another model of experimental colitis. DNBS-induced colitis is a well-defined Th1 based model of transmural inflammation of the colon that may be considered a model of Crohn’s disease. [31], [32] As with DSS-induced colitis, we observed significant reduction in colonic inflammation in IL-13−/− mice following intrarectal challenge with DNBS. The attenuation of DNBS-induced colitis in IL-13−/− mice was observed in macroscopic and microscopic scores as well as MPO activity and pro-inflammatory cytokine production. We also observed a markedly reduced production of 5-HT in IL-13−/− mice. Of note, the 5-HT production observed in DNBS colitis was uniformly lower in all experimental groups compared to levels observed in DSS model. This is most likely due to the differences between the two models of colitis utilized. However, previously established differences in 5-HT content in Th1 and Th2 environments may also underlie such differences observed [15].

In conclusion, we have shown that 5-HT plays a pivotal mediating role in IL-13 driven intestinal inflammation. This study is the first to report an important role of IL-13 in generation of inflammation in two different experimental models (DSS and DNBS) of colitis and also identifies 5-HT as a vital factor in pathogenesis of IL-13-mediated colitis. In consideration with the recent reports of elevated IL-13 levels observed in patients with UC [6], [33] and alterations in EC cells numbers and 5-HT production accompanying different GI disorders, including IBD, [34] the findings of this study shed light on novel immune-endocrine interactions in the gut that may ultimately lead to improved therapeutic strategies in the battle against pathological intestinal inflammation.

## Supporting Information

Figure S1 **Effects of IL-13 deficiency in DSS-induced colitis were not influenced by cages, litters or time.** WT and IL-13−/− mice were given 5% DSS in drinking water for 5 days to induce colitis. All control animals received water without DSS. (A) and (B) Disease activity index (DAI) from two separate experiments evaluating the categories weight loss, stool consistency and feces bleeding demonstrate that the DAI did not differ between cages, litters or time. Data are represented as mean ± SEM from 3 to 5 mice for each experiment; # represents statistically significant difference (p<0.05) between IL-13−/− mice and WT mice administered DSS.
(PDF)

Figure S2 **Effects of IL-13 deficiency in DSS-induced colitis and cell proliferation.** WT and IL-13−/− mice were given 5% DSS in drinking water for 5 days to induce colitis. All control animals received water without DSS. Colonic sections of WT and IL-13−/− mice with or without DSS were immunostained with anti- Ki-67 antibody. Representative micrograph of (A) WT mice post-DSS and (B) IL-13−/− mice post-DSS. (C) Number of Ki-67^+^ cells per gland. * p<0.05; **p<0.01.
(PDF)
