# Supplementary figures and images for: Interleukin 13 and Serotonin: Linking the Immune and Endocrine Systems in Murine Models of Intestinal Inflammation
Source: PLoS One. 2013 Aug 28;8(8):e72774. doi: 10.1371/journal.pone.0072774 (PMC3755966; doi:10.1371/journal.pone.0072774)

**A**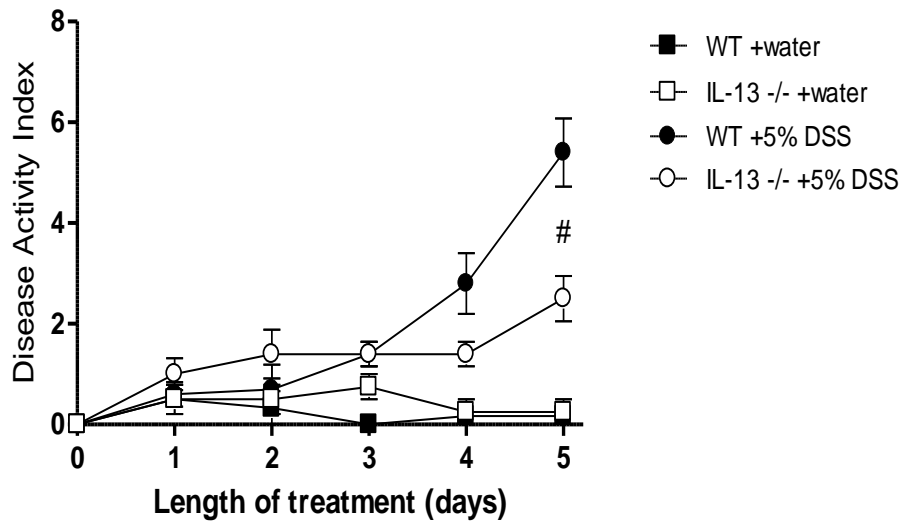**B**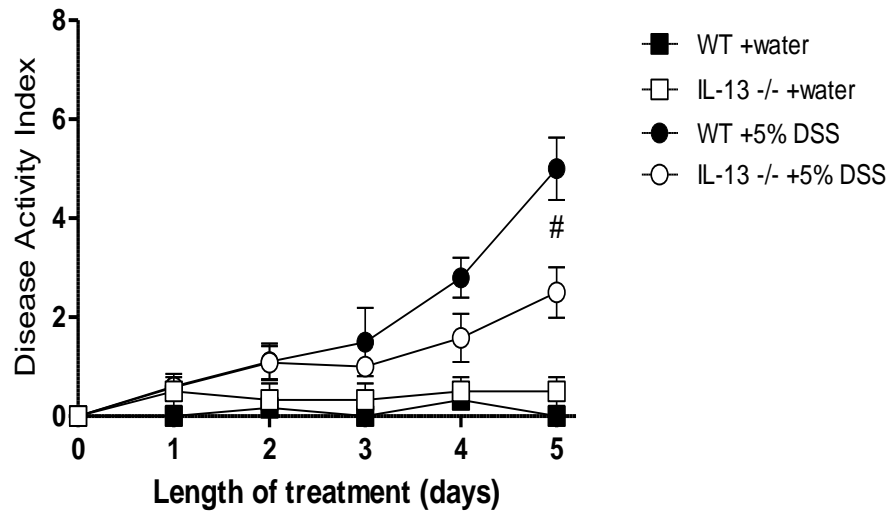

Supplement: Figure S1 — Effects of IL-13 deficiency in DSS-induced colitis were not influenced by cages, litters or time. WT and IL-13−/− mice were given 5% DSS in drinking water for 5 days to induce colitis. All control animals received water without DSS. (A) and (B) Disease activity index (DAI) from two separate experiments evaluating the categories weight loss, stool consistency and feces bleeding demonstrate that the DAI did not differ between cages, litters or time. Data are represented as mean ± SEM from 3 to 5 mice for each experiment; # represents statistically significant difference (p<0.05) between IL-13−/− mice and WT mice administered DSS. (PDF) [file pone.0072774.s001.pdf]

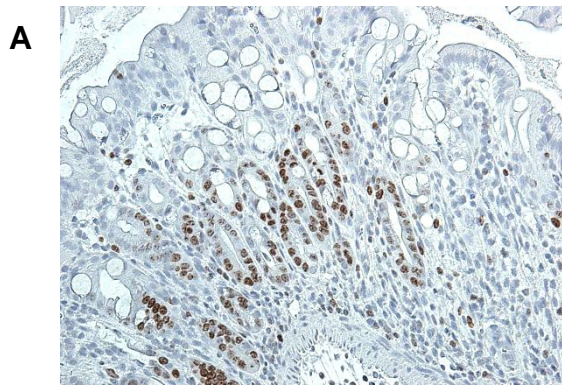

WT +5% DSS

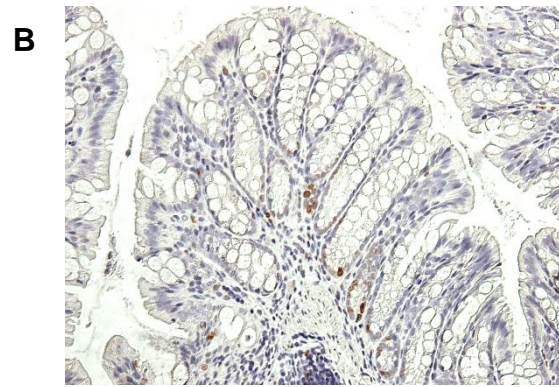

IL-13<sup>-/-</sup> +5% DSS

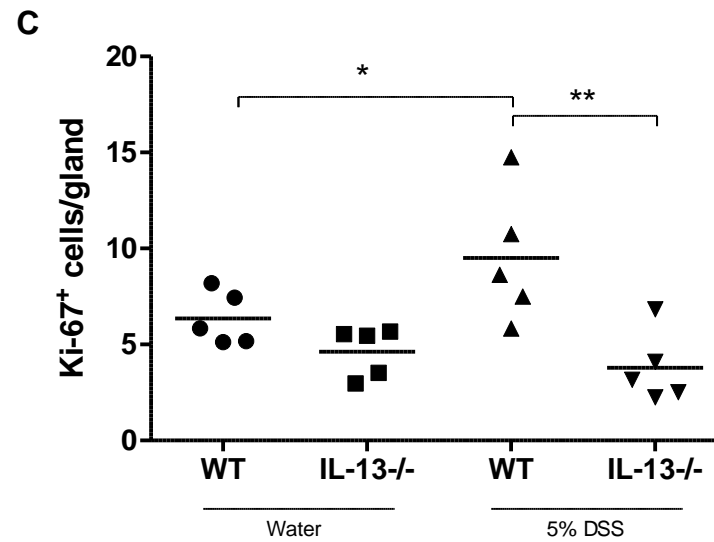

Supplement: Figure S2 — Effects of IL-13 deficiency in DSS-induced colitis and cell proliferation. WT and IL-13−/− mice were given 5% DSS in drinking water for 5 days to induce colitis. All control animals received water without DSS. Colonic sections of WT and IL-13−/− mice with or without DSS were immunostained with anti- Ki-67 antibody. Representative micrograph of (A) WT mice post-DSS and (B) IL-13−/− mice post-DSS. (C) Number of Ki-67+ cells per gland. * p<0.05; **p<0.01. (PDF) [file pone.0072774.s002.pdf]
